# Parkinson's disease and mitophagy: an emerging role for LRRK2

**DOI:** 10.1042/BST20190236

**Published:** 2021-03-26

**Authors:** Francois Singh, Ian G. Ganley

**Affiliations:** MRC Protein Phosphorylation and Ubiquitylation Unit, University of Dundee, Dundee DD1 5EH, U.K.

**Keywords:** autophagy, leucine-rich repeat kinase, mitophagy, Parkinsons disease

## Abstract

Parkinson's disease (PD) is a progressive neurodegenerative disorder that affects around 2% of individuals over 60 years old. It is characterised by the loss of dopaminergic neurons in the substantia nigra pars compacta of the midbrain, which is thought to account for the major clinical symptoms such as tremor, slowness of movement and muscle stiffness. Its aetiology is poorly understood as the physiological and molecular mechanisms leading to this neuronal loss are currently unclear. However, mitochondrial and lysosomal dysfunction seem to play a central role in this disease. In recent years, defective mitochondrial elimination through autophagy, termed mitophagy, has emerged as a potential contributing factor to disease pathology. PINK1 and Parkin, two proteins mutated in familial PD, were found to eliminate mitochondria under distinct mitochondrial depolarisation-induced stress. However, PINK1 and Parkin are not essential for all types of mitophagy and such pathways occur in most cell types and tissues *in vivo*, even in the absence of overt mitochondrial stress — so-called basal mitophagy. The most common mutation in PD, that of glycine at position 2019 to serine in the protein kinase LRRK2, results in increased activity and this was recently shown to disrupt basal mitophagy *in vivo*. Thus, different modalities of mitophagy are affected by distinct proteins implicated in PD, suggesting impaired mitophagy may be a common denominator for the disease. In this short review, we discuss the current knowledge about the link between PD pathogenic mutations and mitophagy, with a particular focus on LRRK2.

## Introduction

A recent report from the United Nations projects that the number of people aged 65 or older, estimated at about 703 million in 2019, will reach almost 1.5 billion in 2050 [1]. As age is the biggest risk factor for neurodegeneration, this maturing of the world population will inevitably lead to a significant increase in the number of patients with neurodegenerative disorders. Parkinson's disease (PD) is a chronic progressive disease and is the second most common neurodegenerative disorder in the world [2]. It affects about 2% of the population over 60 years old, which corresponds today to over 7 million patients. Although symptoms can be relieved by dopamine replacement, there are a lack of treatments that actually alter the course of neurodegeneration [3]. Symptoms were first described in 1817 by James Parkinson in ‘An essay on shaking palsy’ [4], and are characterised by rigidity, bradykinesia, tremor, and postural instability. These symptoms are thought to be due to a loss of striatal dopaminergic (DA) neurons originating in the substantia nigra pars compacta (SNpc), and the accumulation of aggregates of α-synuclein, which are a major constituent of Lewy bodies [5,6]. However, the physiological and molecular mechanisms leading to these phenotypes are still largely unknown. PD is a multifactorial and complex disease that can be subclassified in familial and apparently sporadic PD. While over 90% of cases are sporadic, 22 genes have been identified from familial cases [7]. Many of these PD loci are either directly or indirectly associated with mitochondrial dysfunction [8,9].

Mitochondria are not only in charge of cellular energy production, but also display numerous functions including the production and regulation of reactive oxygen species (ROS), controlling cell death through apoptosis, the regulation of calcium homeostasis, the biosynthesis and metabolism of lipids, the biosynthesis of heme, the regulation of pH within the cell, and much more. These highly specialised and complex organelles form a dynamic network, and their mass is regulated through mitochondrial biogenesis [10] and turnover [11]. In addition to genetics, environmental factors such as exposure to toxins such as MPTP, paraquat, or rotenone (which are all mitochondrial Complex I inhibitors) can lead to the acquisition of Parkinsonian phenotypes [12–15]. Together, this highlights the central role of mitochondria in PD.

Another organelle that has strong links to PD is the lysosome. The lysosome is the end point of many intracellular trafficking pathways and plays a critical function in degrading and recycling a wide variety of cellular components, ranging from single polypeptides to whole organelles, including mitochondria [16]. A large body of work is now emerging that links lysosomal dysfunction to neurodegeneration and in particular PD [17]. Large scale GWAS studies have identified multiple lysosome-related proteins that pose as risk loci for PD [18,19]. Indeed, many lysosomal storage disorders display pronounced neurodegeneration and of relevance here, *GBA1*, the gene that encodes the lysosomal enzyme glucocerebrosidase and is mutated in Gaucher's disease, is a major genetic risk factor for PD [20,21]. Furthermore, rare variants in other lysosomal genes such as *ATP13A2*, *TMEM175*, and *VPS13C* have also been associated with PD [22]. *ATP13A2* (*PARK9*) is a lysosomal P-type ATPase and its loss-of-function mutation causes Kufor–Rakeb syndrome, a juvenile early onset parkinsonism [23]. Transmembrane protein 175 (TMEM175) is a lysosomal potassium channel located in late endosomes and lysosomes, identified in a PD GWAS [24] and its loss-of-function appears to impair autophagy-mediated degradation of α-synuclein [25]. Vacuolar protein sorting-associated protein 13C (VPS13C), is a protein involved in ER-late endosome/lysosome contact sites [26] and its mutations are a monogenic cause of early onset parkinsonism [27].

If mitochondrial or lysosomal dysfunction can lead to PD, then a pathway that involves both these organelles is a prime candidate for further investigation. One such pathway is mitophagy, or the autophagy of mitochondria, which delivers damaged, dysfunctional or superfluous mitochondria to the lysosome for degradation [11]. Multiple mitophagy pathways have been identified that may operate under distinct scenarios. In most tissues, mitophagy occurs under basal conditions as part of normal cellular physiology, the levels of which varies between cell types independently of their mitochondrial content, suggesting the existence of distinct regulatory mechanisms [28–30]. Mitophagy can also occur as a programmed event, as is the case in the late stages of red blood cell maturation [31], cardiomyocyte maturation [32–34], retinal ganglion cell differentiation [35], and in the elimination sperm-derived mitochondria after fertilisation of the egg [36]. Finally, mitophagy can occur in response to a chemical, genetic, or physiologically induced stress and it is these stress pathways that have previously been linked to PD. Our current knowledge on the regulation of mitophagy has been extensively reviewed [5,7,11,37] and here we will focus on the links between pathogenic PD mutations and mitophagy.

## Mitophagy defects in PD

Currently, no core autophagy-essential genes (such as the *ATG* genes) have been directly implicated in PD. However, several PD-linked genes have been identified that modulate mitophagy, which suggests this pathway may be more relevant to PD pathology compared with autophagy in general. As we will describe in the following paragraphs, these PD-related genes could potentially affect mitophagy at different steps and act independently of one another.

**Figure 1.**
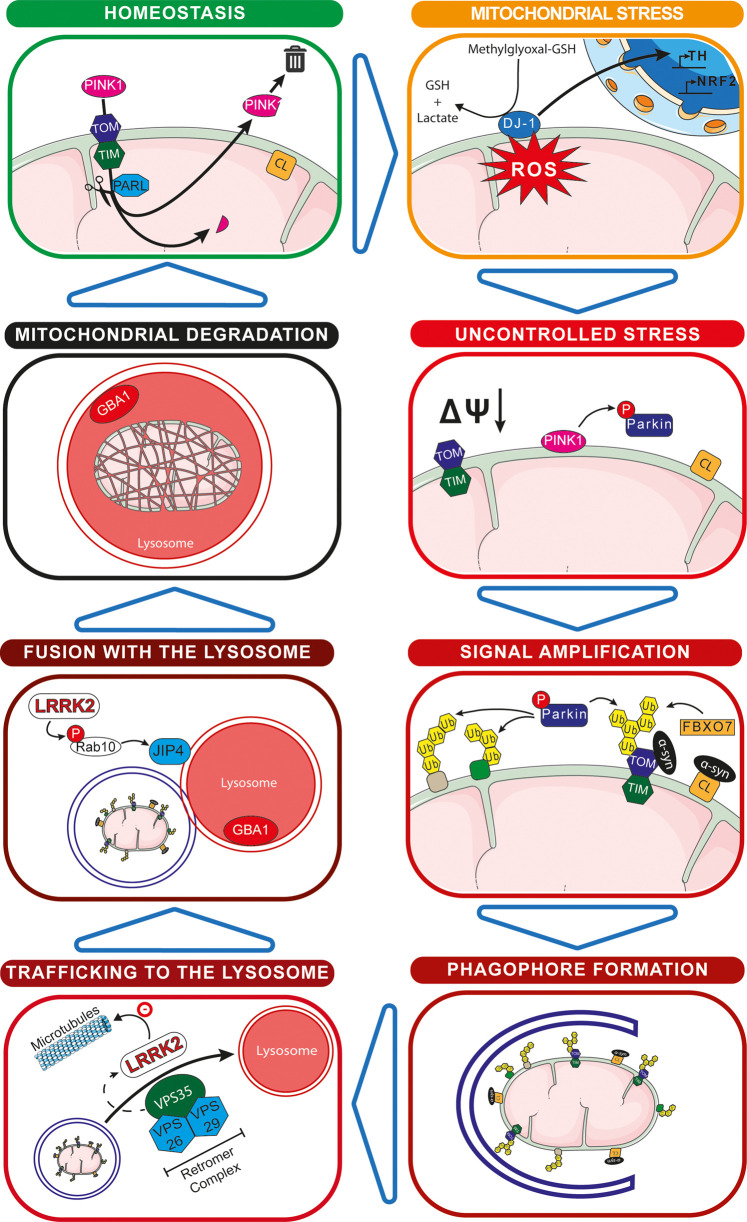
Parkinson's disease-linked genes independently implicated in mitophagy. Homeostasis: PINK1 is imported within the mitochondria where it is cleaved and is then exported for degradation. Cardiolipin is present at the internal mitochondrial membrane where it interacts with the Complex I–Complex III–Complex IV supercomplex. Mitochondrial stress: Oxidized DJ-1 is translocated to the nucleus where it acts as a transcription factor for genes involved in ROS detoxication. Uncontrolled stress: Following loss of mitochondrial membrane potential, PINK1 is stabilized at the surface of the outer mitochondrial membrane where it phosphorylates Parkin. Cardiolipin is translocated to the cytosolic side of the outer mitochondrial membrane. Signal amplification: Activated Parkin and FBXO7 ubiquitylate proteins on the outer mitochondrial membrane. Cardiolipin interacts with α-synuclein to recruit LC3. Phagophore Formation: The amplificated signal recruits the autophagosome machinery that initiates the formation of the phagophore. Trafficking to the lysosome: The newly formed phagosome is trafficked to the lysosome with the help of the retromer complex (VPS35, VPS26, and VPS29). VPS35 enhances the kinase activity of LRRK2. Fusion with the lysosome: LRRK2 phosphorylates Rab10, which in turn promote the translocation of JIP4 to the lysosome to form sensing tubules, enabling the sorting of vesicles. Mitochondrial degradation: The defective mitochondria is degraded in the mitolysosome that contains GBA1.

Most of our current knowledge about mitophagy regulation comes from the comprehensive study of the ubiquitin-dependant pathway of mitophagy that relies on the PD-related PINK1 and Parkin proteins. PTEN induced kinase 1 (PINK1, encoded by the *PARK6* gene) and Parkin (encoded by the *PARK2* gene) are at the heart of the ubiquitin-dependent pathway of mitophagy [3,5,7] and mutation of either results in autosomal recessive forms of PD [38]. *PARK2* was the first gene discovered that directly linked mitophagy and PD in 2008 [39], and *PARK6* was identified soon after, aided by the fact that these two genes are believed to operate in the same pathway [40–44]. This pathway appears to be activated upon extreme mitochondrial stress, with extensive mitochondrial depolarisation being a key trigger. Briefly, PINK1 is a serine/threonine kinase that in normal physiological conditions (Figure 1 — homeostasis), is imported from the cytosol to the mitochondrial matrix thanks to its mitochondria targeting sequence via the TOM/TIM complexes [45,46]. There it is cleaved and inactivated by the protease PARL (presenilin-associated rhomboid-like protein), and the cleaved are then degraded in the cytosol by the proteasome via a n-end rule pathway [3,7,37,47]. Upon mitochondrial damage and loss of mitochondrial membrane potential (Figure 1 — uncontrolled stress), PINK1 acts as a damage sensor and accumulates on the cytosolic side of the outer mitochondrial membrane (OMM) [44]. There, it phosphorylates both ubiquitin and Parkin at their respective Serine 65 residues [48,49]. Sequentially, ubiquitin is first phosphorylated, as the binding of Parkin to pre-existing phospho-ubiquitin has been shown to be required for its phosphorylation [50,51]. Activated Parkin then ubiquitylates multiple OMM targets, leading to an amplification of the ubiquitin signal [11,52] (Figure 1 — signal amplification). This signal leads to the recruitment of the autophagy machinery, via ubiquitin-binding autophagy receptors such as optineurin and NDP52 [53], and the engulfment of the mitochondrial fragment into a mitophagosome. Concomitant fusion with a lysosome results in mitochondrial degradation and recycling of its constituent components [54]. The PINK1/Parkin pathway has been extensively studied *in vitro*, often with harsh chemical uncouplers that are at odds with normal physiological conditions. Thus, it has not been clear as to when this pathway may be activated *in vivo*. Indeed, it was recently shown that this pathway does not seem to affect basal mitophagy in both mice and *Drosophila* [55–57]. Hence, the prevailing idea is that the PINK1/Parkin pathway constitutes a stress pathway in response to distinct mitochondrial toxins and pathological conditions, the identity of which are currently unclear.

It should be noted that aside from mitophagy, PINK1 and Parkin have also been involved in additional modes of mitochondrial turnover that may also be relevant for PD. For example, PINK1/Parkin-mediated proteasomal turnover of OMM proteins in *Drosophila* not only stimulates mitophagy, but also promotes the selective turnover of a subset of mitochondrial electron transport chain subunits [58]. Additionally, in mammalian cells, the PINK1/Parkin pathway has been involved in the generation of mitochondria-derived vesicles (MDVs) in response to oxidative stress, triggered by uncouplers such as CCCP or oligomycin and antimycin A, which are selectively delivered to late endosomes/lysosomes independently of the mitophagy pathway [59,60]. In a more physiological setting, heat stress in PINK1 knockout primary bone-marrow-derived macrophages increases the mitochondrial antigen presentation (MitAP) of 2-oxoglutarate dehydrogenase through MDVs [61]. Of importance to PD, it was recently shown that intestinal bacterial infection in PINK1 knockout mice induced MitAP and provoked a CD8+ T cell infiltration into the brain that lead to reduced DA neuron axon varicosities and motor impairments [62]. These data highlight a potential cross-talk between mitochondrial quality control and autoimmune mechanisms in the aetiology of PD, an area where LRRK2 has also been implicated [63].

Despite PINK1 and Parkin dominating the mitophagy field, other genes involved in PD have recently been described that influence mitochondria and mitophagy (see Figure 1). Mutations in DJ-1, encoded by the *PARK7* gene, cause recessive forms of PD [64] and it acts as a redox sensor that localises to mitochondria [7]. Oxidation of DJ-1 on Cysteine 106 leads to its translocation into the nucleus where it functions as a transcriptional co-activator and corepressor [65,66] (Figure 1 — mitochondrial stress). Notably, it positively regulates the expression of tyrosine hydroxylase, the enzyme responsible for the conversion of tyrosine to L-DOPA, and it also regulates the expression of NRF2, a master regulator of the anti-oxidative stress response [65]. *In vitro*, DJ-1 showed a methylglyoxal-adduct hydrolase activity, which could protect low-molecular thiols such as Coenzyme A, highlighting its role in redox homeostasis [67]. Although the localisation of this protein is controversial, pathogenic DJ-1 mutants have been reported to translocate from the cytosol to the mitochondrial matrix *in vitro* [68]. Also, DJ-1 deficiency is associated with the age-dependant relocalisation of OMM hexokinase 1 to the cytosol, which in turn inhibits the PINK1/Parkin pathway [69]. Importantly, loss of DJ-1 leads to oxidative stress within the cell, mitochondrial fragmentation, and potentially impaired mitophagy [64,70].

As mentioned above, one of the histopathological hallmarks of PD is the presence of Lewy bodies [6], a major constituent of which is aggregates of α-synuclein. α-Synuclein, encoded by the *PARK1* gene, is a small soluble protein mostly expressed in the brain. Though the function of this protein is not clear, it may play a role in neurotransmitter release [71]. Pathogenic α-synuclein has been shown to bind preferentially to mitochondria [72], in particular to Translocase of the outer mitochondrial membrane 20 (TOMM20), which inhibits mitochondrial protein import leading to mitochondrial dysfunction [73]. Evidence also suggests α-synuclein may indirectly affect mitophagy by interacting with Cardiolipin [74], or up-regulating the expression of Miro, an OMM GTPase regulating mitochondrial movement [75]. Of relevance, it was recently shown that when cardiolipin translocates from the inner mitochondrial membrane to the OMM, it interacts with α-synuclein and can recruit LC3 to the mitochondria to trigger mitophagy [76] (Figure 1 — signal amplification).

Mutations in the *FBXO7* gene (*PARK15*) encoding for F-box only protein 7 (FBXO7) causes autosomal recessive early onset PD. FBXO7 has been shown to ubiquitinate TOMM20 [77] (Figure 1 — signal amplification), and to act in a common mitophagy pathway with PINK1 and Parkin, with overexpression of human FBXO7 in Drosophila rescuing a Parkin loss-of-function phenotype [78].

Mutation in the *VPS35* gene (*PARK17*), encoding for the Vacuolar protein sorting-associated protein 35 (VPS35), causes autosomal dominant late-onset PD [7]. VPS35 is a key component of the retromer cargo recognition complex and thus regulates endocytic membrane trafficking, which can influence autophagy in general [79] (Figure 1 — trafficking to the lysosome). Relevantly, it has been reported to be a Parkin substrate and in turn, its ubiquitylation regulates retromer-mediated endocytic sorting, including that of the autophagy-essential protein ATG9A [80]. Furthermore, the PD-related D620N mutation of VPS35 was recently shown, in patient-derived neurons, to cause α-synuclein accumulation and mitochondrial defects, which was attributed to impaired autophagy [81]. Of note, The D620N mutation has been shown in both mouse and human to be an upstream regulator of LRRK2 kinase activity [82].

As previously mentioned, homozygous mutations in the *GBA1* gene cause the lysosomal storage disorder Gaucher disease [83]. However, heterozygous *GBA1* mutations are also risk factors for developing PD [20], increasing the risk of disease development by about 21-fold [84]. While it is estimated that between 7% and 12% of patients with PD carry a mutation in the *GBA1* gene, only a minority of *GBA1* mutation carriers actually develop PD symptoms [20]. Given the lysosomal-related function of GBA1, mutations provoke general autophagy defects that would also be predicted to disrupt mitophagy [85]. Additionally, mice with the GBA1 knock-in mutation L444P have been reported to have impaired mitochondrial function and mitophagy [86] (Figure 1 — mitochondrial degradation).

## LRRK2 and basal mitophagy

**Figure 2.**
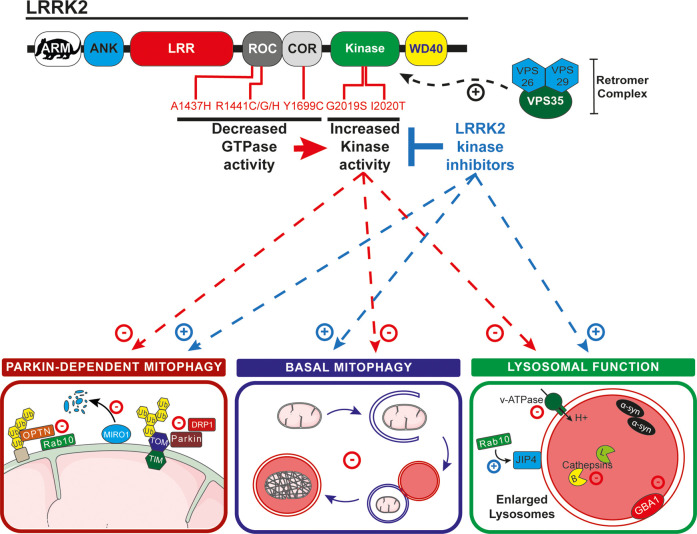
Roles of LRRK2 in mitophagy. Schematic of LRRK2 domain structure is shown at the top and pathogenic mutations in the catalytic domains are noted. VPS35, a retromer subunit, enhances LRRK2 kinase activity. Parkin-dependent mitophagy: Increased LRRK2 activity negatively impacts PINK1/Parkin-dependent mitophagy by reducing PARKIN interaction with the TOMM complex and DRP1, as well as reducing Rab10 recruitment to mitochondria and the interaction between optineurin (OPTN) with ubiquitylated OMM proteins. Basal mitophagy: LRRK2 kinase activity also impairs basal mitophagy and lysosomal function (which impacts the end point of mitophagy). Lysosomal function: At the lysosome LRRK2 regulates sorting via Rab10 phosphorylation and JIP4 and it negatively regulates the function of GBA1. It also impacts the vacuolar ATPase, leading to a higher lysosomal pH, which in turn impacts cathepsin-mediated degradation of α-synuclein. LRRK2 kinase inhibitors attenuate many of these effects, for example, inhibitors such as GSK3357679A, rescues the mitophagy defects in mice carrying the pathogenic G2019S LRRK2 mutation.

Coding variants in the leucine-rich repeat kinase 2 (*LRRK2*, *PARK8*) gene cause an autosomal dominant form of PD and are responsible for the majority of familial cases [87]. LRRK2 is a large (286 kDa) multidomain protein consisting of an armadillo (ARM) domain, an ankyrin domain (ANK), a leucine-rich repeat domain (LRR), a Ras of complex GTPase domain (ROC), a C-terminal of ROC domain (COR), a kinase domain, and a WD40 domain (Figure 2). Coding variants in LRRK2 leading to PD segregate to its catalytic core, such as in the GTPase domain (R1441C/G/H or Y1699C) and in its kinase domain (G2019S and I2020T). The G2019S mutation being the most frequent genetic cause of PD, representing 4–5% of familial cases and about 1% of sporadic cases [88]. All the known pathogenic mutations of LRRK2 lead to an increased kinase activity [89]. Given the track record of small molecule kinase inhibitors as effective drugs for other diseases, LRRK2 kinase inhibitors offer considerable and obvious potential. Indeed, small molecule LRRK2 kinase inhibitors, as well as an antisense LRRK2 oligo, are currently in clinical trials for PD indications [90]. In addition, other types of inhibitors have been developed, either targeting the G-protein cycle by inhibiting its GTPase activity or by inhibiting LRRK2's protein–protein interactions (such as with 14-3-3) [91]. The proliferation in LRRK2 inhibitor development highlights that pharmacologically targeting LRRK2 could be a highly potent solution to diminish pathogenic LRRK2 effects.

The fact that LRRK2 is a large multidomain protein suggests that it has numerous functions within the cell. However, its physiological roles are still poorly understood, as are the aspects of its functions that are relevant for PD. In-line with the hypothesis that mitochondrial and lysosomal dysfunction underlie PD, an emerging body of data supports a key role for LRRK2 at these organelles. Work has shown that patient-derived fibroblasts from G2019S carriers display abnormal mitochondrial morphology and function [92]. Likewise, this same LRRK2 mutation has been shown to increase mitochondrial DNA damage in human-derived cells [88,93]. The abnormal mitochondrial phenotype is also present in mice with aged G2019S knock-in animals displaying hallmarks of impaired fission and altered dynamics [94,95]. Mutations of LRRK2 have also been shown to alter lysosomal function (Figure 2 — lysosomal function). In various *in vitro* and *in vivo* models of the G2019S mutation, enlarged lysosomes were reported, suggesting a strong link between LRRK2 and lysosomal function [96]. A recent *in vitro* study with neuronal primary cultures identified LRRK2 as an interactor with the a1 subunit of the Vacuolar ATPase [97]. In that article, the authors show that the R1441C mutation, but not the G2019S, leads to an increased lysosomal pH and consequently a decreased lysosomal function, though this effect appears independent of the kinase activity of LRRK2. In contrast, it was also reported that the G2019S mutation modestly alters lysosomal morphology and acidification, and that this effect is dependent on the kinase activity of LRRK2 [98]. Here, the defect in lysosomal function disrupted Cathepsin B and L mediated turnover and led to an accumulation of insoluble α-synuclein. Furthermore, LRRK2 kinase activity has also been reported to reduce GBA1 activity in neurons derived from patients, through its substrate Rab10, and this effect was dependent on LRRK2 kinase activity [99]. Additionally, LRRK2 kinase activity plays a key role in phagosome maturation and lysosomal trafficking [100] and recent work has shown LRRK2 interacts with the motor protein adaptor JIP4 at lysosomes to regulate lysosomal tubulation in a process termed LYTL (lysosomal tubulation/sorting driven by LRRK2 [101]) (Figure 1 — fusion with the lysosome, and Figure 2). In relation to intracellular trafficking and the cytoskeleton, LRRK2 PD mutants have previously been shown *in vitro* to display a higher microtubule association compared with WT LRRK2 [102]. This was recently confirmed with the high-resolution structure of the catalytic portion of LRRK2, showing microtubule binding through its WD40 domain [103]. Lastly, microtubule-based signalling and ciliogenesis was disrupted in LRRK2 R1441C knock-in mice, which displayed decreased ciliation in cholinergic neurons in the striatum [104]. Taken together, these data suggest an important link between LRRK2 and microtubules, which in turn could have consequences for mitochondria and lysosomes.

Given the published evidence of pathogenic LRRK2 playing a role in both mitochondrial and lysosomal dysfunction, it follows that recent work also implicates impaired mitophagy. Research has linked the LRRK2 pathway with that of PINK1/Parkin-dependent mitophagy [105–108] (Figure 2 — Parkin-dependent mitophagy). For example, LRRK2 was shown to form a complex with Miro, which is required for its efficient removal during PINK1/Parkin-dependent mitophagy [108]. Impaired mitochondrial dynamics were also observed in cell lines co-expressing Parkin and LRRK2. Expression of LRRK2 G2019S disrupted Parkin-dependent mitophagy, potentially via reducing Parkin's interaction with OMM proteins, including the fission regulating GTPase DRP-1 [106]. Work has also shown in PD-derived patient fibroblasts that Rab10, a downstream substrate of LRRK2, could recruit the mitophagy receptor optineurin [105]. Impaired mitophagy was also observed in fibroblasts derived from patients with the G2019S mutation [107]. These data are summarised in Figure 2.

The above evidence suggests that impaired stress-induced Parkin-dependent mitophagy could be a contributing factor to PD pathology. However, we have previously shown that mitophagy occurs *in vivo* in the absence of overt stress, under so-called basal conditions, in a manner that is independent of PINK1 and Parkin [55,56]. It is reasonable to assume that both impaired stress-induced mitophagy and impaired basal mitophagy can lead to the accumulation of dysfunctional mitochondria and reduced cellular health. If so, then compromised basal mitophagy could also be an important contributing factor for PD development. This may well be the case, as we recently showed that LRRK2 kinase activity inversely correlates with basal levels of mitophagy in specific organs and cell types *in vivo* (Figure 2 — basal mitophagy). Using mitophagy (*mito*-QC) and autophagy (*auto*-QC) reporter mice we were able to show that the common pathogenic G2019S LRRK2 mutation, which results in increased kinase activity, reduced basal mitophagy in cells and tissues of clinical relevance, including midbrain DA neurons and microglial cells [109]. In contrast, the absence of LRRK2 resulted in increased basal mitophagy in these same cell types. Interestingly, this phenomenon was not observed in all cell types, implying cell, and tissue-specific consequences for LRRK2 activity. Importantly though, using a next-generation LRRK2 inhibitor, GSK3357679A, we were able to correct the mitophagy defect in LRRK2 G2019S-expressing mice and restore mitophagy to wild type levels in both DA neurons and microglia. The effect of LRRK2 on mitophagy potentially appears to be selective, as general macroautophagy seems to be largely unaffected both *in vitro* [105] and *in vivo* [109]. This suggests a significant disruption of lysosomal function is not occurring.

A major question remaining concerns how LRRK2 kinase activity regulates basal mitophagy (and indeed how it regulates other cellular processes). It is not yet clear whether LRRK2 activity plays a direct mitophagic role via regulating autophagy machinery, or more indirect through disruption of mitochondrial movement/function or membrane trafficking in general. However, a subset of Rab GTPases were recently identified to be key substrates of the LRRK2 kinase [110]. Rab GTPases are master regulators of vesicular trafficking and thus ideally placed to regulate and influence auto-lysosomal pathways [87]. Their interaction with LRRK2 has been extensively reviewed recently [89,111,112]. Notably, LRRK2 has been shown to phosphorylate Rab12 [110,113] and SQSTM1/p62 *in vitro* [114]. Rab12 is a positive regulator for autophagy that regulates mTORC1 activity, a sensor for nutrient availability and key inhibitor of starvation-induced autophagy [115,116]. Additionally, Rab12 has also been shown to directly interact with autophagosomes and influence their trafficking [117,118]. p62 was the first autophagy cargo receptor described in mammals and is thought to be important in regulating the incorporation of cellular components into forming autophagosomes [119,120]. Thus, LRRK2 substrates are known to regulate autophagy but further work is needed to determine if, and how, their regulation by LRRK2 impacts this process.

## Conclusions

PD is a complex neurodegenerative disorder and, as with many such diseases, its pathophysiology and molecular mechanisms are still poorly defined. However, these molecular details are slowly being revealed and there is a gathering optimism within the field. Though several pathogenic gene mutations have been associated with defects in mitochondrial elimination, whether mitophagy itself plays an active role in PD pathology remains to be proven. The emergence of the discovery of new links between PD-associated mutations and mitophagy defects imply that impaired mitochondrial elimination might indeed be a key event in PD aetiology. Though a better appreciation of these pathways is still required, enhancing mitophagy pharmacologically could hold great potential for PD treatment.

## Perspectives

- Ageing of the population will lead to the emergence of neurodegenerative disorders such as PD.
- Altered mitophagy appears to be a common impairment between many pathogenic PD mutations, suggesting a potential key role.
- A better understanding of mitophagy pathways might be key in the future of understanding the aetiology of Parkinson's disease and its potential treatment.

## Abbreviations

DA: dopaminergic
FBXO7: F-box only protein 7
LRR: leucine-rich repeat domain
MDVs: mitochondria-derived vesicles
MitAP: mitochondrial antigen presentation
OMM: outer mitochondrial membrane
PD: Parkinson's disease
PINK1: PTEN induced kinase 1
TOMM20: translocase of the outer mitochondrial membrane 20
